# High Spatial-Resolution Digital Phase-Stepping Shearography

**DOI:** 10.3390/jimaging7100192

**Published:** 2021-09-27

**Authors:** Awatef Rashid Al Jabri, Kazi Monowar Abedin, Sheikh Mohammed Mujibur Rahman

**Affiliations:** Physics Department, College of Science, Sultan Qaboos University, P.O. Box 36, Muscat 123, Oman; s109932@student.squ.edu.om (A.R.A.J.); mujib@squ.edu.om (S.M.M.R.)

**Keywords:** digital shearography, speckle interferometry, phase-stepping, high spatial resolution, center-loaded plate, non-destructive testing

## Abstract

Digital phase-stepping shearography is a speckle interferometric technique that uses laser speckles to generate the phase map of the displacement derivatives of a stressed object, and hence can map the stresses of a deformed object directly. Conventional digital phase-stepping shearography relies on the use of video cameras of relatively lower resolution, in the order of 5 megapixels or lower, operating at a video rate. In the present work, we propose a novel method of performing high spatial resolution phase stepping shearography. This method uses a 24 megapixel still digital imaging device (DSLR camera) and a Michelson-type shearing arrangement with an edge-clamped, center-loaded plate. Different phase-stepping algorithms were used, and all successfully generated shearograms. The system enabled extremely high-resolution phase maps to be generated from relatively large deformations applied to the test plate. Quantitative comparison of the maximum achieved spatial resolution is made with the video-rate cameras used in conventional shearography. By switching from conventional (video) imaging methods to still imaging methods, significantly higher spatial resolution (by about 5 times) can be achieved in actual phase-stepping shearography, which is of great usefulness in industrial non-destructive testing (NDT).

## 1. Introduction

Digital shearography, or digital phase-stepping shearography, has become an established interferometric tool in industry for measuring the whole-field deformation derivatives of test objects and for non-destructive testing [1,2,3,4,5,6]. In this technique, based on laser speckle interferometry, usually one of the mirrors in a sheared Michelson-type arrangement is moved by predetermined amounts to perform the necessary phase-stepping of the light beam, and capturing interference images of the test object at each step [1,2]. The phase map of the displacement derivatives of the deformed object can be constructed by performing the necessary computations between the interference images captured before and after the deformation is applied on the test object. In conventional shearography, a video camera, operating at a video rate, is used to capture the sheared images of the test object. The maximum resolution of the video camera used is of the order of 5 megapixels [7,8,9,10]. This limits the spatial resolution for the generated shearograms and phase maps and, consequently, limits the maximum value of the deformations that can be successfully observed.

In the present paper, we present a novel and unconventional method of performing digital phase-stepping shearography with high-resolution still imaging devices. Previously, we used an 18 megapixel digital single-lens reflex camera (DSLR) to generate subtraction-mode shearograms of a test object [11]. No phase-stepping was performed, and an inherent limitation of the camera (discussed later) limited the spatial resolution of the subtraction-mode shearograms generated. In the present work, we used a different, higher resolution DSLR camera (which does not have this limitation) and used it in different, phase-stepping algorithms to generate phase maps in a variety of ways. All algorithms successfully produced phase maps of excellent quality and resolution. Maximum deformations measured successfully were of the order of 55 microns (about 90 times the wavelength of light used) under the given experimental conditions.

## 2. Materials and Methods

**Figure 1:**
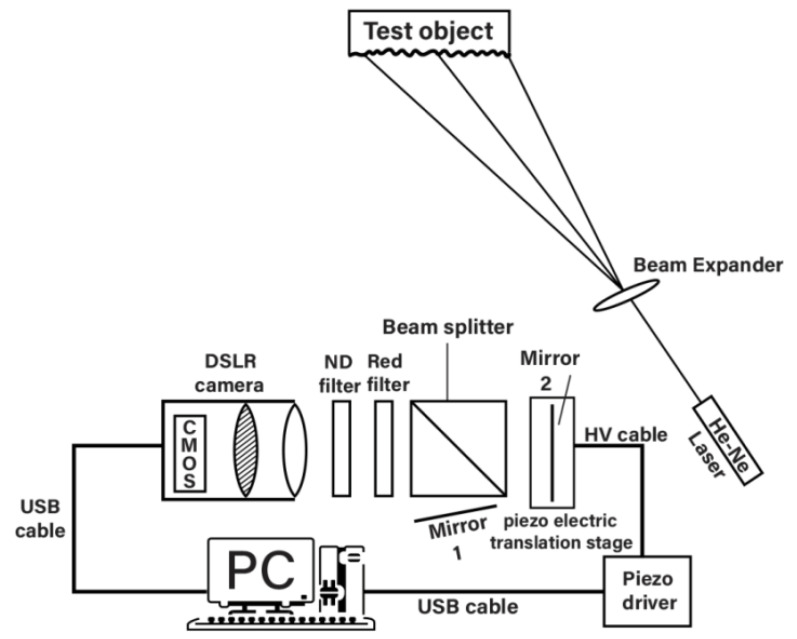
Schematic diagram of the shearographic system.

The schematic diagram of the experiment is shown in Figure 1. Light from a He–Ne laser (wavelength 633 nm) was expanded by a 40X microscope objective and allowed to fall on a test object. The He–Ne laser was used for its superior beam quality. The light scattered by the test object was incident on a modified Michelson interferometer, placed about 300 mm away. One of the mirrors of the interferometer was tilted, either vertically or horizontally, by a given amount to produce a known amount of image shear in either the horizontal or vertical direction. The sheared images were captured by the digital still camera (DSLR), which was a Nikon Model D5300 with a 25 megapixel CMOS image sensor. The CMOS image sensor has dimensions of 23.5 mm × 15.6 mm, producing a native pixel resolution of 6000 × 4000 pixels in the maximum resolution mode. The pixel size on the sensor is 3.9 μm × 3.9 μm. The camera was attached to an AF-P DX Nikkor zoom lens of 18–55 mm focal length. The camera has no internal optical (spatial) low-pass filter (OLPF) [12], which may produce a blurring effect on the images. A 620 nm red glass filter was used to cut out most of the ambient light, and a neutral-density (ND) filter was used to control the amount of light entering the camera and to prevent any saturation of the image sensor. The camera was directly connected to a desktop IBM PC, via a USB cable without any image grabber hardware, and all the various camera functions, including shutter operation, exposure setting, aperture setting, etc., could be controlled from the PC, using the software *ControlMyNikon*. In these experiments, the camera was mostly operated in the aperture-priority mode selected by the mode dial of the camera, which allowed us to control the speckle size on the camera sensor. The camera then selected the necessary shutter speed and exposure settings automatically. The autofocus function was disabled, and manual focus mode was selected.

Before performing the main experiments, we first measured the speckle size on the captured image on the image sensor, and compared it to the expected theoretical value. We covered one the mirrors in the Michelson system (e.g., mirror 2), so that the light from only the other mirror enters the camera. We then captured an image for each value of the *F*-number (focal length/aperture diameter of the imaging lens) selected by the camera. We expanded each captured image by Photoshop, identified the individual speckles, and measured the sizes of a number of speckles in pixels. Since the pixel size on the image sensor is known (3.9 μm), we could estimate the average speckle size (in microns) for each value of the *F*-number selected. The theoretical speckle size is given by the equation [2]:

(1) $$\Delta S=\frac{\lambda f}{D}\left(1+M\right)=\lambda F\left(1+M\right).$$

where *λ* is the wavelength of light, *f* and *D* are the focal length and aperture diameter of the imaging lens, respectively, and *M* is the magnification of the imaging system.

**Figure 2:**
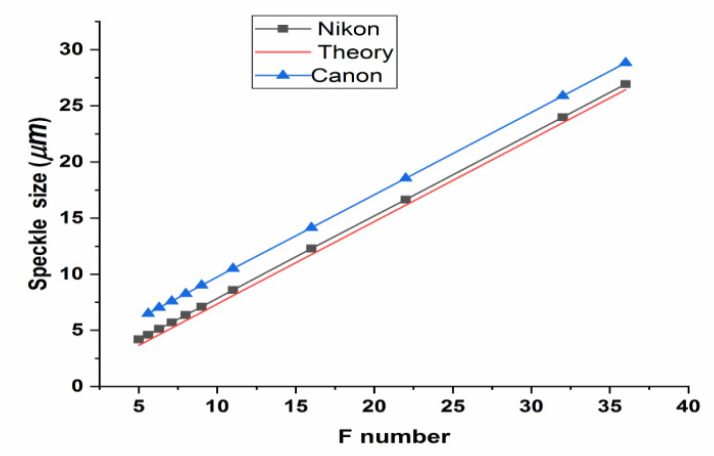
Observed average speckle size, as a function of *F*-number for the two DSLR cameras. The red line indicates the theoretical speckle size.

The plot of observed average speckle size is shown as a function of the *F*-number in Figure 2. The theoretical speckle size (from Equation (1)) is shown as a continuous straight line for a measured value of M = 0.16. For comparison, the measured speckle size for another DSLR camera (Canon EOS 100D with 18 megapixel CMOS sensor) is also shown in the same graph. There is a good agreement between the theoretical and measured speckle size for Nikon D5300. On the other hand, the considerable difference between theoretical and measured speckle sizes for the Canon EOS 100D camera shows that speckles are significantly larger than expected, and the internal (spatial) optical low-pass filter (OLPF) [13] used in this camera effectively increases the speckle size that can be observed, due to the associated blurring effect produced by it, by suppressing the sharper (high-frequency) details of the speckles. This certainly will limit the maximum resolution that can be attained in a shearography experiment using this camera. For almost all of the present experiments, we selected a *F*-number of 5.6. This results in a speckle size of about 4.6 microns, compared to a pixel size of 3.9 microns for the Nikon D5300 camera. This means that the camera is operated in the sub-Nyquist domain, where the Nyquist criterion is not satisfied. More explanation about this aspect is included in the Discussion section.

**Figure 3:**
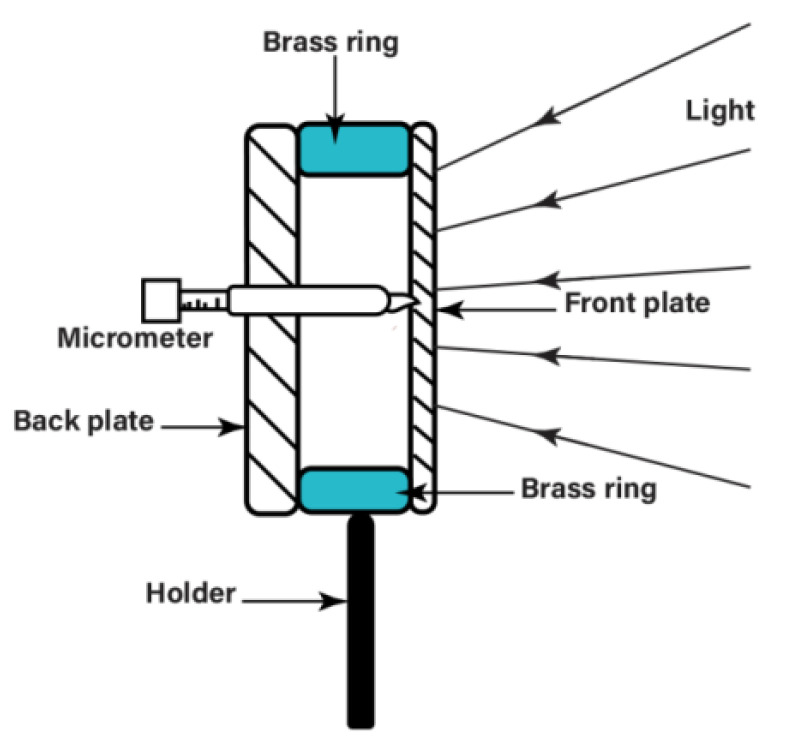
Details of the test object used to produce known deformations (modified from Abedin, et al., Proc. SPIE 2019, 11100, 1110001-7, Ref. [11]).

The test object was a circular aluminum plate about 1 mm thick and about 80 mm in diameter (Figure 3). It was bolted on the circular brass ring symmetrically around the periphery with 12 M4 steel bolts. A thick aluminum back plate, about 5 mm thick, was mounted on the other side of the brass ring with bolts, and a small micrometer was installed and fixed on this thick plate through a hole drilled at its center. The tip of the micrometer pushed against the thin front plate, and by rotating the micrometer, a small, measurable amount of deformation could be applied to the front aluminum test plate. The estimated accuracy of deformation measurements was about 5 μm. This plate was sprayed with white paint to improve the reflectivity, and also to make it optically rough. All the components of the system, including the test object, were rigidly mounted on an optical breadboard, without any active vibration isolation being used. In the particular experiments described below, an image shear of about 1.2 mm was applied to mirror 1.

For phase-stepping, mirror 2 was mounted on a piezo-electrically driven translation stage (Thorlabs NFL5DP20 (Newton, NJ, USA), and the position of the mirror 2 was precisely controlled by the piezoelectric controller (Thorlabs model KPZ101 Piezo driver (Newton, NJ, USA)). The piezoelectric controller was connected to the PC by a USB cable and was fully controlled by the supplied software. We determined from the manufacturer’s data that a piezo voltage of about 0.3 V was required to move the mirror 2 by *λ*/8 (*λ* = 633 nm), thus producing a phase shift of 90 deg for the light beam reflected from mirror 2. A 120 deg phase shift (*λ*/6 mirror movement) at the same wavelength required a piezo voltage of about 0.4 V.

**Figure 4:**
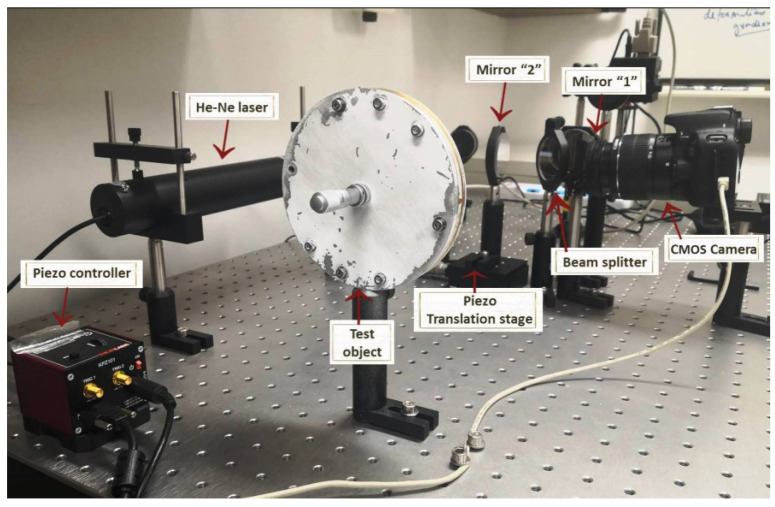
Photograph of the assembled digital phase-stepping shearography system.

The photograph of the assembled system is shown in Figure 4. After the system was assembled and adjusted, a number of speckled images were captured by the camera. Between each image, the mirror 2 was moved by the appropriate amount determined by the particular phase-stepping algorithms being used. A given amount of deformation was then applied to the center of the test surface by rotating the micrometer, and another series of images were captured by the camera after the deformation were applied. Between each image, the mirror 2 was moved by the appropriate amount, also determined by the particular phase-stepping algorithms being used. All the images (in compressed JPEG form) were immediately transferred to the hard disk of the PC by the USB cable. The image capture and transfer typically take about a couple of hundred milliseconds.

The following six phase-stepping algorithms were used in the experiment:

(a) 4 + 4, (b) 4 + 1, (c) 4 + 2, (d) 3 + 3, (e) 3 + 1, (f) 3 + 2

The first number refers to the images captured before the deformation is applied to the test object, and the second number refers to the number of images captured after the deformation is applied. The details of these algorithms can be found in Ref. [2]. All the required phase-stepping calculations between images were performed using programs written in MATLAB.

In our present experiments, the 4 + i algorithms required a step size (mirror movement) of *λ*/8 (79 nm at *λ* = 633 nm), while the 3 + i algorithms required a step size of *λ*/6 (105 nm at *λ* = 633 nm).

## 3. Results

**Figure 5:**
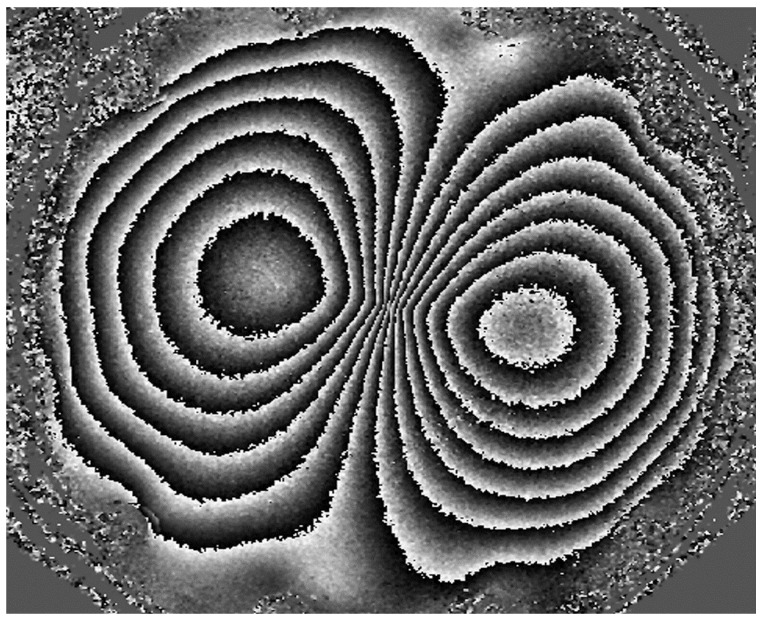
Phase map generated by the 4 + 4 phase stepping algorithm for a 55 μm central deformation for the Nikon D5300 camera.

We used the Nikon camera to generate most of the images in digital phase-stepping shearography. We first applied different amounts of central deformation to the test object, and we generated phase maps corresponding to each deformation. The phase map corresponding to a 55 μm central deformation of the test plate using the 4 + 4 phase stepping algorithm is shown in Figure 5. The raw phase map was filtered using the sine-cosine filtering technique [1]. A large number of fringes of excellent contrast can be observed in the phase map, with high density clear fringes at the center.

**Figure 6:**
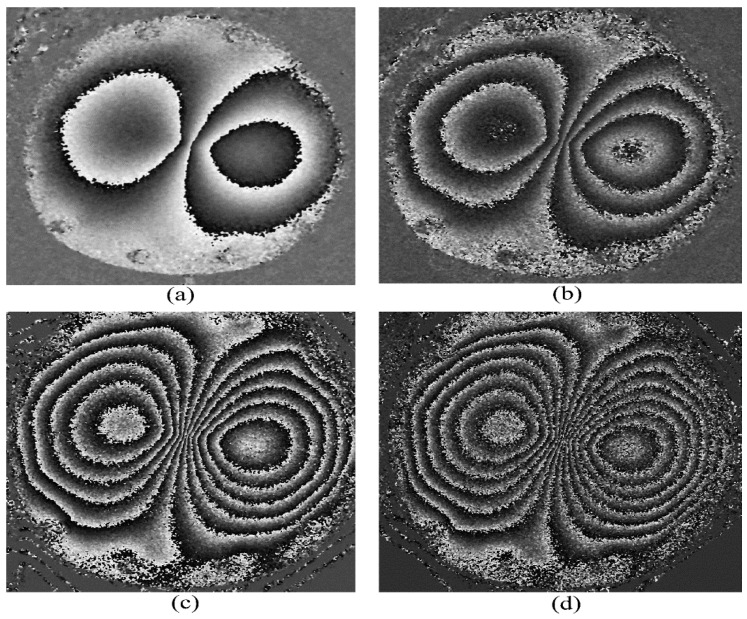
Phase map generated by the 4 + 1 phase stepping algorithm for applied central deformations of (**a**) 10 μm, (**b**) 25 μm, (**c**) 45 μm, and (**d**) 55 μm from the Nikon D5300 camera.

The filtered phase maps corresponding to various amounts of central deformations using the 4 + 1 algorithm are shown in Figure 6. As the deformation is increased, more and more fringes appear, as expected. All the fringes have good contrast, even for the larger deformations. The minimum measurable deformation under these conditions is approximately 5–10 μm, and the maximum measurable deformation with acceptable contrast at the central region is roughly about 55 μm. Larger deformations failed to produce phase maps of acceptable quality and contrast.

**Figure 7:**
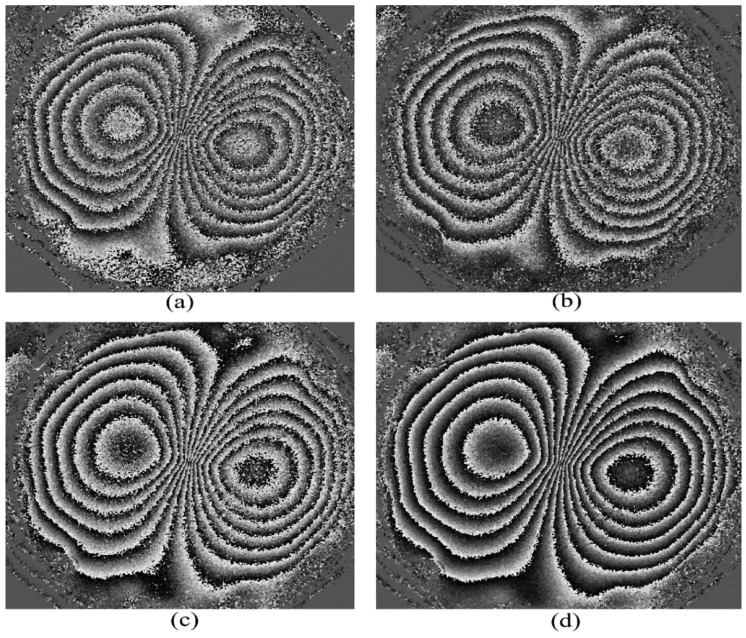
Phase map generated for a 55 μm central deformation by different phase-stepping algorithms from the Nikon D5300 camera, (**a**) 4 + 1, (**b**) 3 + 1, (**c**) 3 + 2, and (**d**) 3 + 3.

We then applied a maximum central deformation of 55 μm to the test plate and generated filtered phase maps individually for the different 4 + 1, 3 + 1, 3 + 2, 3 + 3, and phase-stepping algorithms mentioned above. This is shown in Figure 7. We were able to generate phase maps successfully for all of the algorithms we tried, even for a deformation as large as 55 microns. All the images have acceptable contrast, even for relatively high-density fringes near the center. Larger deformations (e.g., 70 microns) failed to produce phase maps of acceptable contrast.

**Figure 8:**
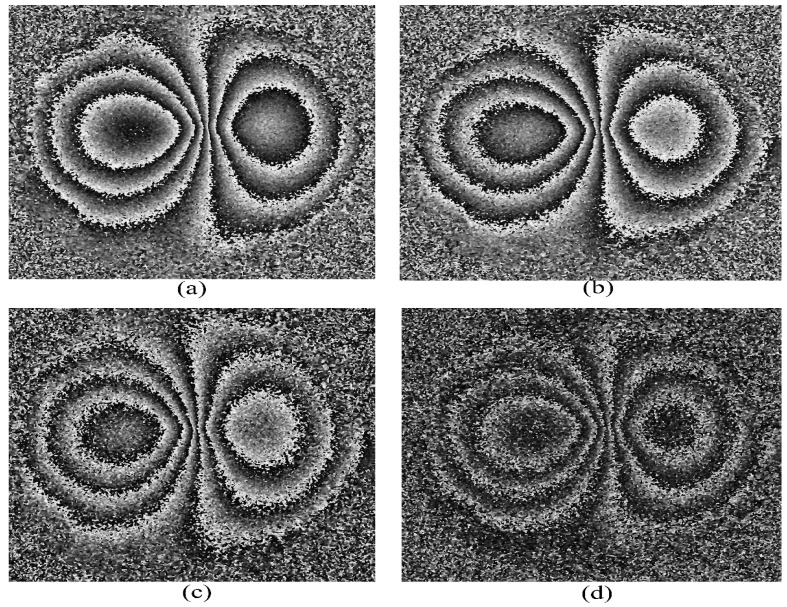
Phase map generated for a 20 μm central deformation by the Canon EOS 100D camera for different phase-stepping algorithms, (**a**) 4 + 4, (**b**) 3 + 3, (**c**) 3 + 2, and (**d**) 3 + 1.

**Figure 9:**
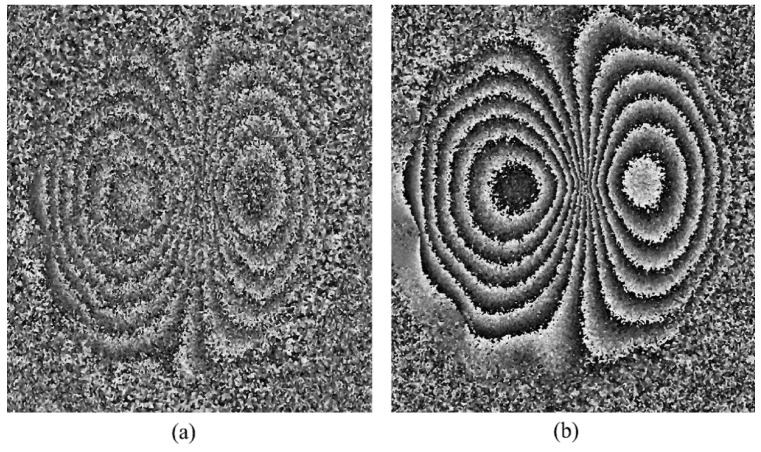
Phase maps generated for a 30 μm central deformation by the Canon EOS 100D camera for different phase-stepping algorithms, (**a**) 4 + 1 and (**b**) 4 + 4.

For comparison, the filtered phase maps generated from the other camera (Canon EOS 100D), with 18 megapixel resolution and with the integral low-pass filter, are shown in Figure 8 for different phase-stepping algorithms. The applied deformation is 20 microns. Larger deformations failed to produce useful phase maps for all the algorithms except the 4 + 4 and 4 + 1. For the last two algorithms, we were able to generate phase maps for a maximum 30-micron central deformation. These are shown in Figure 9. Deformations larger than 35 microns failed to generate any phase maps at all.

## 4. Discussion

The above results show that it possible to generate phase maps of deformations of good quality if an imaging device of high effective spatial resolution (e.g., 25 megapixels) is used. This higher resolution was achieved by abandoning the use of conventional video-imaging methods altogether, and switching to the still imaging methods capable of significantly higher resolutions. Still images were captured only on command and *only when required*. The effective resolution is considerably greater than the conventional video cameras used in conventional shearography experiments used so far (of the order of 5 megapixels). In the present experiment, phase maps were generated for a large deformation of 55 μm under the given experimental conditions (for 1.2 mm image shear), and a large number of high-density fringes could be observed with good contrast. The density of the observed fringes was higher than that observed in the literature (e.g., the shearograms in Figure 5.1.8 and 6.1.3 in Ref. [1] and Figure 4.2 and 5.2 in Ref. [2]). In our experiments, the ability to observe a large number of fringes in the phase map implies a greater spatial resolution. Phase maps of reasonable quality were produced for all algorithms, namely, 4 + 4, 4 + 1, 3 + 1, 3 + 2, and 3 + 3.

To have an estimate of the improvement of resolution achieved by the still imaging methods, compared to the conventional video imaging methods used so far, we proceed as follows. In any speckle measurement system, the ultimate spatial resolution achieved is limited by the speckle noise or speckle size. We can control the speckle size by controlling the aperture of the camera (Equation (1)). If we choose a wider aperture, the speckle size will be smaller. The smallest useful speckle size would be roughly equal to the pixel size of the sensor. Now, if the fringe separation in the shearogram or phase map approaches the speckle size in a given experiment, the fringes will no longer be discernible (the fringes will be effectively “lost” in the speckle noise). If, as a rough rule of thumb, we take the minimum usable fringe separation to be about 5 times the average speckle size, then the minimum useful fringe separation ∆*fr* on the sensor is:

(2) Δfr=5ΔS=5Δpix

where ∆*pix* is the pixel size. Assuming that we have two cameras (one having a 24 megapixel sensor and one having a 5 megapixel sensor), and assuming they have the same aspect ratio and sensor size (this condition, however, is not satisfied in all cases), then the ratio of the pixel sizes of the two cameras (24 megapixel camera vs. 5 megapixel camera) is, approximately:

(3) $$\frac{\Delta pix\left(24MP\right)}{\Delta pix\left(5MP\right)}=\surd \frac{5}{24}=\frac{1}{2.2}$$

It means that the minimum useful fringe separation ∆*fr* for the 24 megapixel camera is about 2.2 times smaller than the 5 megapixel camera. Consequently, the useful lateral spatial resolution (which is inversely proportional to ∆*fr*) should also be about 2.2 times greater for the 24 megapixel camera, compared to the 5 megapixel camera. The increase in resolution is effective in both horizontal and vertical directions. The “*areal resolution*” (resolution in terms of areas) will be increased by the square of this factor, i.e., by a factor of 4.8. Of course, if we can use a higher resolution camera (e.g., 100 megapixels, see later in this discussion), then the spatial resolution will be correspondingly higher as well.

It has been pointed out by Zhu et al. [14] that phase maps produced by the 3 + 1 algorithms have lower quality, compared to those produced by 3 + 2 and 3 + 3 algorithms. Similarly, it was stated by Yang and Xie [2] (p. 65) and Zhao et al. [15] that 4 + 1 algorithms generate poor phase maps compared to other algorithms, such as 4 + 2. In our experiments, we were able to generate phase maps of acceptable quality, even for large deformations for both 4 + 1 and 3 + 1 algorithms. The phase maps produced by 3 + 1 and 4 + 1 algorithms were only of slightly lower quality, compared to those produced by 3 + 2 or 3 + 3 algorithms (see Figure 7).

For the F number selected (F = 5.6), the Nikon camera operation did not satisfy the Nyquist criterion, which requires that the ratio of the speckle size to the pixel size (N) be N = 2. In our case, it was about N = 1.2, which implies that the camera was operated in the sub-Nyquist domain. The selection of this F-number was made after some preliminary experiments. We also performed shearography experiments with higher F-numbers, such as F = 9 (for F = 9, N is approximately 1.9, very close to the Nyquist criterion). We were able to successfully generate phase maps for these higher values of F, where the speckles were larger in relation to the pixel size, and therefore, more closely approximate the Nyquist criterion. However, we found out that even if we were able to generate phase maps, the raw phase maps appeared to be noisier. So, we settled for a F-number of F = 5.6, which gave us the best overall performance.

For applications such as nondestructive evaluation and testing, it is desirable to generate the phase maps with the smallest number of images, especially after the deformation is applied. It enables phase maps to be generated in the smallest possible time, and to generate phase maps in situations where the deformation is changing dynamically. From this viewpoint, 3 + 1 and 4 + 1 algorithms are the most desirable, since they require a single image to be taken after the deformation is applied, without the need for any further mirror displacement and image acquisitions.

Our success in generating phase maps for both 4 + 1 and 3 + 1 algorithms manifests that they can be used for nondestructive testing applications, as well as for quantitative measurements, provided a digital camera of high enough spatial resolution is used. It is true that with the digital still cameras of the current generation, a couple of milliseconds are required to capture and transfer the image to the computer, and the *live-fringe technique*, which captures deformation in real time, is difficult to implement in this situation. Moreover, to perform real-time processing of phase maps, the processing speed of the computer becomes a limiting factor, and the problem becomes more serious as the image size is increased. However, it should be possible to measure and quantify slowly changing deformations without much difficulty, especially if the process is automated. The present camera can capture images at the rate of five frames per second, so it should be possible, at least by post-processing, to generate phase maps of deformations at this temporal resolution using either the 4 + 1 or 3 + 1 algorithms.

The Canon EOS 100D camera, in general, produced phase maps of inferior quality (e.g., Figure 9a), compared with the Nikon D5300 camera. The reasons, in our opinion, are, (1) lower spatial resolution of the Canon EOS 100D camera (18 megapixels, compared to 24 megapixels for the Nikon camera) and (2) the presence to the optical low-pass filter in front of the imaging device (COMS sensor), which prevented the full spatial resolution (18 megapixels) of the camera to be utilized. This is supported by the data in Figure 2, which shows that the observed speckle size for the Canon EOS 100D is indeed larger than that predicted by theory, and significantly larger than the speckle sizes from the Nikon D5300 camera.

Low-pass filters are used in some cameras to reduce the Moire effect [16] in normal photographic applications, for example, when capturing certain repetitive details in photographing everyday objects (such as in shirts, curtains, etc.) which exceeds the camera resolution. The trend in recent higher-resolution cameras is to avoid using the low-pass filter entirely, as was the case with the Nikon D5300 camera.

All the images generated by both cameras use the well-known JPEG format, which involves some image compression. This format uses a discrete cosine transform (DCT) and some truncation of high frequency components, thus discarding some information from the original image (lossy compression) [17]. The effect of JPEG image compression on digital holographic data has been discussed by Darakis and Soraghan [18] The effect of image compression on general fringed images has been investigated by Harvey et al. [19]. The compression process in JPEG enables files of smaller sizes to be generated from the camera. For example, for the Nikon D5300 camera, the uncompressed image (6000 × 4000 pixels) from the camera should have file size of 24 megabytes, but the JPEG image files generated by this camera have an average file size of about 4 megabytes. This represents a significant amount of lossy compression, with a compression ratio of about 6. As shown in the present experiments, this loss of data does not destroy the essential information which is encoded in the image by the intensity distribution in the laser speckles, and, furthermore, does not place any impediment in the complex calculation of phase data from the lossy JPEG images generated by the cameras. It appears that the high-frequency components of the speckle data, which are discarded, do not play a significant role in the calculation of the phase maps in our case, at least with compression factors as large as 6.

Another advantage of the digital still cameras, compared to the conventional video cameras, is simpler interfacing with the computer, without requiring any image grabber hardware at all. Interfacing is simply performed by plugging the USB cable from the camera to the USB port of the PC. The use of compressed JPEG images conserves memory space as well.

In principle, any high-resolution digital imaging device (either DSLR or mirrorless type) can be used in this application, provided that the aperture can be controlled manually. Recently, digital still cameras are available with a native resolution of about 100 megapixels [20,21]. It should be possible to use these imaging devices in any shearographic application, with the advantages of significantly higher spatial resolutions that could be achieved. To give just an example, this may enable one to permit more detailed observations of smaller defects in a large surface in a nondestructive testing application. Future improvements in the still camera sensor resolutions promise to achieve even higher spatial resolutions in digital shearography in the near future.

In spatial phase shift shearography, which uses only two images (reference image and measuring image) to generate phase maps of deformations, the spatial resolution is severely limited by the spatial resolution of the imaging device [2,7]. The higher spatial resolution of the imaging device, such as the one used in the present experiment, can be expected to significantly improve the performance of spatial phase shift shearography. Spatial phase shifting is also very suitable to capture dynamic deformations, because only two images are required for complete phase map generation. The increased spatial resolution should, in principle, also enable higher sensitivity of detection of smaller defects in nondestructive testing applications, as compared to cameras of lower resolutions.

In summary, the novelty of the present method, compared to the previous methods are the following: (a) achievement of much higher spatial resolution (about 5 times or more), (b) use of lossy compressed images for successful phase map generation, (c) generation of excellent quality phase maps using the more challenging but useful 3 + 1 and 4 + 1 phase shifting algorithms, (d) possibility of using it in extremely high-resolution NDT experiments, and (e) possibility of using it in high-resolution spatial phase shifting shearography experiments for dynamic deformation measurements.

## 5. Conclusions

In conclusion, we can say that we have generated phase maps of significantly higher spatial resolution (by about 5 times) by using a higher-resolution still imaging method, compared to the video imaging method used in conventional video-rate phase-stepping shearographic systems. The higher resolution still imaging device enables one to generate good quality phase maps from relatively large deformations using a variety of phase-stepping algorithms, permitting more accurate observations and more sensitive NDT applications. The method can be applied, in principle, to almost any digital imaging device capable of taking high-resolution still pictures. Moreover, it could be used in spatial phase shift shearography to achieve higher overall performance, as well.
